# Characterization of Walking in Mild Parkinson’s Disease: Reliability, Validity and Discriminant Ability of the Six-Minute Walk Test Instrumented with a Single Inertial Sensor

**DOI:** 10.3390/s24020662

**Published:** 2024-01-20

**Authors:** Gaia Bailo, Francesca Lea Saibene, Virginia Bandini, Pietro Arcuri, Anna Salvatore, Mario Meloni, Anna Castagna, Jorge Navarro, Tiziana Lencioni, Maurizio Ferrarin, Ilaria Carpinella

**Affiliations:** 1 IRCCS Fondazione Don Carlo Gnocchi, 20148 Milan, Italy; gbailo@dongnocchi.it (G.B.); fsaibene@dongnocchi.it (F.L.S.); vbandini@dongnocchi.it (V.B.); parcuri@dongnocchi.it (P.A.); asalvatore@dongnocchi.it (A.S.); acastagna@dongnocchi.it (A.C.); jnavarro@dongnocchi.it (J.N.); tlencioni@dongnocchi.it (T.L.); icarpinella@dongnocchi.it (I.C.); 2 Neurology Unit, Azienda Ospedaliero-Universitaria, 09123 Cagliari, Italy; ma.meloni@aoucagliari.it

**Keywords:** Parkinson’s disease, gait, inertial sensors, rehabilitation assessment

## Abstract

Although the 6-Minute Walk Test (6MWT) is among the recommended clinical tools to assess gait impairments in individuals with Parkinson’s disease (PD), its standard clinical outcome consists only of the distance walked in 6 min. Integrating a single Inertial Measurement Unit (IMU) could provide additional quantitative and objective information about gait quality complementing standard clinical outcome. This study aims to evaluate the test–retest reliability, validity and discriminant ability of gait parameters obtained by a single IMU during the 6MWT in subjects with mild PD. Twenty-two people with mild PD and ten healthy persons performed the 6MWT wearing an IMU placed on the lower trunk. Features belonging to rhythm and pace, variability, regularity, jerkiness, intensity, dynamic instability and symmetry domains were computed. Test–retest reliability was evaluated through the Intraclass Correlation Coefficient (ICC), while concurrent validity was determined by Spearman’s coefficient. Mann–Whitney U test and the Area Under the receiver operating characteristic Curve (AUC) were then applied to assess the discriminant ability of reliable and valid parameters. Results showed an overall high reliability (ICC ≥ 0.75) and multiple significant correlations with clinical scales in all domains. Several features exhibited significant alterations compared to healthy controls. Our findings suggested that the 6MWT instrumented with a single IMU can provide reliable and valid information about gait features in individuals with PD. This offers objective details about gait quality and the possibility of being integrated into clinical evaluations to better define walking rehabilitation strategies in a quick and easy way.

## 1. Introduction

Parkinson’s Disease (PD) represents a fast-growing neurodegenerative condition attributed to the loss of dopaminergic neurons in the substantia nigra. The disease is characterized both by motor symptoms, which include tremors, rigidity, bradykinesia/akinesia and postural instability, and by non-motor symptoms, such as cognitive impairment, sleep disorders, sensory abnormalities and fatigue [1]. These movement abnormalities are the hallmark of PD, and with the progression of the disease, they can deeply affect the quality of life, leading to high disability in the activities of daily living [2]. Motor disorders in PD can be assessed with a wide range of clinical rating scales and tests [3], which includes the 6-Minute Walk Test (6MWT) among the recommended evaluation tools [4]. The 6MWT is a simple and non-invasive test introduced by the American Thoracic Society (ATS) to evaluate the biological responses of all the systems involved in the walking exercise, by measuring the distance a subject can walk in 6 min [5]. It was initially developed for patients with moderate to severe heart or lung disease, but over time has been extended as a reliable method to estimate physical functional capacity in post-stroke subjects, subjects with Alzheimer’s disease, and subjects with multiple sclerosis [6,7,8]. Regarding PD, the test was described as highly trustworthy and clinically useful for motor assessment [9,10]. Nevertheless, objective gait features are lacking in the standard clinical 6MWT, since the only outcome measure provided is the walked distance [5]. As support to clinicians, different technologies can be integrated to obtain quantitative information and provide additional insights into the walking pattern.

Inertial measurement units (IMUs) are wearable devices containing accelerometer and gyroscopic sensors, characterized by lightness, ease of use and relative cost-effectiveness, therefore enabling extensive motor assessment and overcoming conventional restrictions of laboratory measurements. These devices have been validated as reliable and accurate instruments to detect gait spatiotemporal characteristics (e.g., cadence, stride length, stance and swing phase duration) in healthy people, people with multiple sclerosis and persons with PD [11,12,13,14,15], during short walking tests such as the 10-m walk test and the timed 25-foot walk test. Given their ease of use even in larger environments, IMUs have been integrated also into long walking tests (i.e., the 2-min walk test and the 6MWT) which allow the analysis of both straight-line walking and turning, the latter not assessed during short tests. Moreover, compared to short tests, the availability of several longer walking bouts permits to computing of not only common spatiotemporal parameters, but also more advanced gait quality metrics (i.e., symmetry, variability/regularity, smoothness, instability) usually requiring a high number of strides [16]. This, in turn, allows to extract valuable gait information from different populations, ensuring a more comprehensive gait assessment without further burdening the subject [17,18]. Regarding the test–retest reliability of the above metrics during long walk tests, this has been analyzed in healthy subjects, people with multiple sclerosis and individuals post-stroke with overall good results [19,20,21]. In contrast, to the best of our knowledge, no such analysis has been performed on people with PD so far.

Although the above-cited studies and most of those summarized in a recent review [17] follow a multiple-IMU approach, the application of a single inertial sensor has been explored due to its easiness and quickness of use in clinical settings. The agreement between a single IMU and a reference system (i.e., sensorized mats or optoelectronic systems) has been already assessed for the measurement of spatiotemporal parameters in healthy adults and subjects with PD [22,23,24]. Moreover, while gait variability and spatiotemporal metrics have demonstrated good reliability during the 6MWT in elderly and persons post-stroke [25,26], this aspect has not yet been studied in PD.

Therefore, the present study has three aims: (i) to evaluate the test–retest reliability of a set of spatiotemporal gait parameters and gait quality metrics obtained by a single IMU positioned on the lower trunk of individuals with mild PD during the 6MWT, (ii) to assess the concurrent validity of these metrics with clinical measures, and (iii) to analyze their ability to discriminate between healthy subjects and people with mild PD.

## 2. Materials and Methods

### 2.1. Participants

Twenty-two persons with a diagnosis of Parkinson’s Disease were recruited at IRCCS Fondazione Don Carlo Gnocchi Onlus, Milan, Italy. The inclusion criteria were: age between 50 and 85 years; PD diagnosis according to MDS Criteria [27]; ability to walk 30 m with or without an assistive device; ability to comprehend and sign an informed consent; modified Hoehn and Yahr (mH&Y) score between 1 to 2.5; stable pharmacological treatment in the last four weeks. The exclusion criteria were: vascular, familiar and drug-induced forms of parkinsonism, other known or suspected causes of parkinsonism (metabolic, brain tumor, etc.) or any suggestive features of atypical parkinsonism; significant comorbidities and/or severe systemic diseases that would preclude exercise participation (e.g., recent surgery; unstable cardiac dysfunction; anemia; hepatosis; pulmonary disorders; chronic renal failure; auditory, visual and/or vestibular dysfunctions; presence of deep brain stimulation); previously diagnosed psychiatric diseases; dementia as defined by Montreal Cognitive Assessment (MoCA Test) Correct Score < 15.51 [28]. During the period of the study, participants maintained the scheduled therapies and medications. Moreover, all tests were performed during the “on” phase (phase in which the optimal drug therapy and motor improvement were achieved in the subject). 

Ten healthy subjects (HS) with comparable sex and age to the PD population were also involved in the study as control group. Inclusion criteria were age above 18 years and healthy general condition. Exclusion criteria were any neurological, musculoskeletal or cardiopulmonary disorders that might affect gait. 

The total sample size (22 people with PD and 10 HS) was considered adequate based on previous studies finding a 6MWT distance (mean ± standard deviation) of 499 m ± 85 m in healthy subjects of 70–79 years old [29], and of 316 m ± 142 m in persons with PD [9], resulting in an effect size of 1.56. These data indicated that 28 subjects (19 people with PD and 9 HS) were necessary to obtain a difference between groups with α = 0.05, power = 0.95 and an allocation ratio of 0.5. The sample size of the PD group was chosen considering the worst-case scenario of an Intraclass Correlation Coefficient (ICC) of 0.5 (minimum ICC value for fair reliability [30]), with two observations per subject, power = 80% and α = 0.05.

All the study subjects signed a written informed consent before participating that was approved by the local Ethical Committee (code: 13/2023/CE_FdG/FC/SA).

### 2.2. Clinical Assessment

Subjects with PD underwent a clinical examination prior to the first instrumented test session. The administered clinical scales and tests are described in Table 1.

### 2.3. Instrumented Assessment

The test–retest instrumented assessment was performed at 24–72 h intervals, maintaining the same testing conditions. Before starting both sessions, the 6MWT was explained to the subject following the ATS guidelines [5]. Subjects walked for 6 min at a sustainable speed between two delimiter cones placed at a 30 m distance in a straight hallway, free of obstacles and other people. If needed, they could use walking aids, slow down and stop and resume walking as soon as possible. 

**Figure 1:**
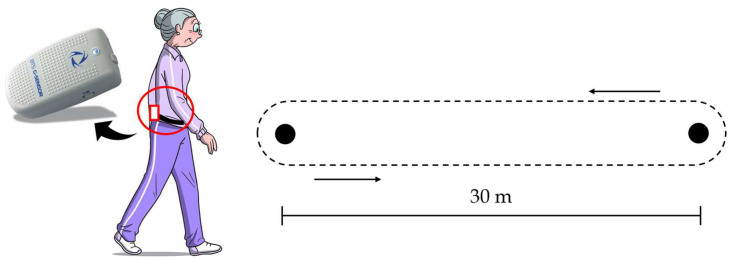
Positioning of G-Walk sensor and gait protocol for test–retest sessions.

Raw lower trunk accelerations and angular velocities data were acquired at a 100 Hz frequency with the G-Walk (BTS Bioengineering, Milan, Italy), placed on the patient’s skin projection of vertebra L5 and held by a semi-elastic belt (Figure 1). G-Walk is a single IMU measuring 70 × 40 × 18 mm with a weight of 37 g, consisting of a triaxial accelerometer 16 bit/axes (±2 g), a triaxial gyroscope 16 bit/axes (±2000 °/s) and a triaxial magnetometer 13 bit (±1200 uT). The system is equipped with Bluetooth 3.0 connection to transmit acquired data to a tablet PC. The frequency of 100 Hz was considered adequate for our study since it is within the range of sampling rates (25–1000 Hz) used in previous studies [17,37].

### 2.4. Data Processing

**Figure 2:**
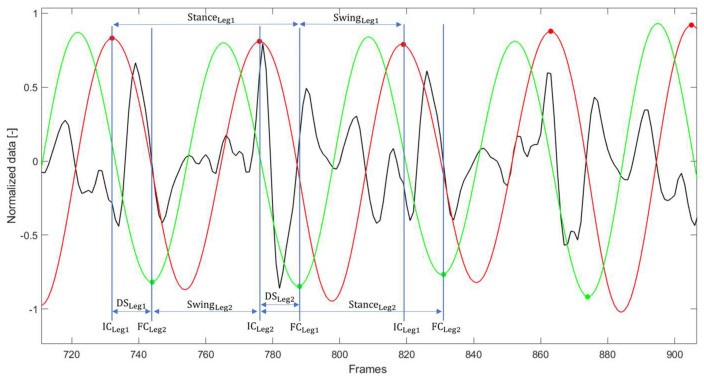
Examples of raw vertical acceleration (black), integrated wavelet (red) and integrated and differentiated wavelet (green). Initial contacts (IC) correspond to the red dots, final contacts (FC) correspond to the green dots. Stance durations, swing durations and double supports (DS) of both legs are represented. Signals are reported after being normalized for their maximum value for graphical representation only.

Data processing was performed using MATLAB R2022b (MathWorks, Natick, MA, USA). To analyze separately each straight hallway, signals were divided according to the turns detected by the IMU [38]. Due to the subject body shape and any incorrect sensor applications, the alignment of the device may present with offset. For this reason, trunk accelerations along the three axes (anteroposterior (AP), mediolateral (ML) and vertical (VT)) were reoriented to a horizontal–vertical coordinate system following the widely used method of Moe-Nilssen [39], where the AP axis was positive in the forward direction, ML axis was positive in the left direction, and VT axis in the upward direction. To estimate heel-strike (initial contact, IC) and toe-off (final contacts, FC) events within the gait cycle, the filtered VT acceleration (4th order low-pass Butterworth filter with a cut-off frequency of 10 Hz) was integrated and differentiated using a continuous wavelet transform (CWT). Following the algorithm defined by McCamley et al. [40] and optimized by Del Din et al. [41] for the validation on people with PD, according to current axis directions, IC events were identified as the frames corresponding to the maxima of the integrated signal, and with further CWT differentiation, FC events were identified as frames corresponding to the minima of the obtained signal, as shown in Figure 2. Only data related to ten consecutive steady-state strides (20 steps) in the middle of each hallway were considered to determine gait features. A set of 41 metrics was computed from trunk acceleration and angular velocity components. The metrics, organized in gait domains as previously proposed [42,43,44], are listed in Table 2.

All parameters were obtained for each hallway and then averaged for each subject’s total number of hallways executed during the 6MWT.

### 2.5. Statistical Analysis

Statistical analyses were performed using SPSS software (version 28, SPSS Inc., Chicago, IL, USA). Participants’ gender and age were compared using Pearson’s chi-square test and Mann–Whitney U test, respectively. Since most of the investigated features exhibited a non-normal distribution at the Shapiro–Wilk test, non-parametric tests were conducted. The level of significance was taken at 5%.

Between-day test–retest reliability was evaluated through the Intraclass Correlation Coefficient (ICC). ICC was computed with a 95% confidence interval, using a two-way mixed-effect, absolute agreement, multiple measurements model. An ICC lower than 0.49 was classified as poor, between 0.50 and 0.74 as fair, between 0.75 and 0.89 as good, and an ICC greater than 0.90 as excellent [30]. Additionally, the Standard Error Measurement (*SEM*) and the Minimal Detectable Change (*MDC*) at 95% confidence were calculated for each metric. The *SEM* provides a value for measurement error in the same units as the measurement itself, and was calculated with Equation (1):

(1) $$SEM={SD}_{mean}\cdot \;\sqrt{1-ICC}$$

where *SD* corresponds to the mean of the standard deviations obtained from data in test and retest sessions. The *MDC* represents an estimate of the slightest change in a measure that can be detected objectively. Change scores larger than *MDC* indicate real change, while change scores lower than *MDC* indicate a variation due to measurement error and/or within-subject variability. It was determined with Equation (2) [52]:

(2) $$MDC=1.96\cdot \sqrt{2}\;\cdot SEM$$

Concurrent validity of the instrumented metrics was investigated using Spearman’s rank correlation coefficient ($r_{s}$) between clinical scales and gait parameters that showed good/excellent reliability (i.e., ICC ≥ 0.75). In particular, correlation analyses were performed between all reliable metrics and clinical measures of motor impairment (H&Y and MDS-UPDRS III) and gait and balance (6MWT, TUG, PIGD, Akinesia/Bradykinesia). Moreover, for a subset of instrumented features (variability/regularity, jerk, intensity and symmetry metrics), the association with specific clinical scales (e.g., Tremor, Rigidity, Asymmetry) was also analyzed, on the basis of the reasonable hypothesis that they could measure similar constructs. Absolute values of $r_{s}$ between 0.2 and 0.4 indicate a small correlation, between 0.4 and 0.6 moderate correlation, between 0.6 and 0.8 strong correlation and between 0.8 and 1 very strong correlation [53]. 

The ability of the instrumented metrics to discriminate PD subjects from healthy people was evaluated by the Mann–Whitney U test with Benjamini–Hochberg (B-H) correction for multiple comparisons (false rate of 5%) [54], where a *p*-value lower than 0.05 was reputed statistically significant. Moreover, the Area Under the Receiver Operating Characteristic (ROC) curve (AUC) was computed. Values lower than 0.7 represent a poor discriminant ability, between 0.7 and 0.8 moderate discriminant ability, and values greater than 0.8 good discriminant ability [55]. The discriminant ability was assessed only for valid metrics, i.e., those exhibiting a significant correlation with at least one clinical measure. 

## 3. Results

### 3.1. Participants Description

Demographic and clinical characteristics of People with PD (PwPD) and healthy subjects (HS) are reported in Table 3. Age, sex distribution and Body Mass Index were comparable between groups. The 6MWT clinical score showed a statistically significant difference between PwPD and HS, with the former group showing reduced walking endurance. By contrast, the TUG test was comparable between groups.

### 3.2. Test–Retest Reliability

ICCs and 95% confidence intervals of investigated instrumented features for people with PD are represented in Table 4, as well as SEM and MDC. Table 4 shows also the mean values of test and retest sessions of the gait parameters.

Thirty-nine out of forty-one variables exhibited good/excellent reliability between sessions with an ICC ≥ 0.75, while the normalized RMS of AP acceleration and the Harmonic Ratio (HR) in the ML direction were measured with fair reliability (ICC ≥ 0.50). The 6MWT clinical score showed excellent reliability with an ICC of 0.98, consistent with values found in the literature [9].

### 3.3. Concurrent Validity

**Figure 3:**
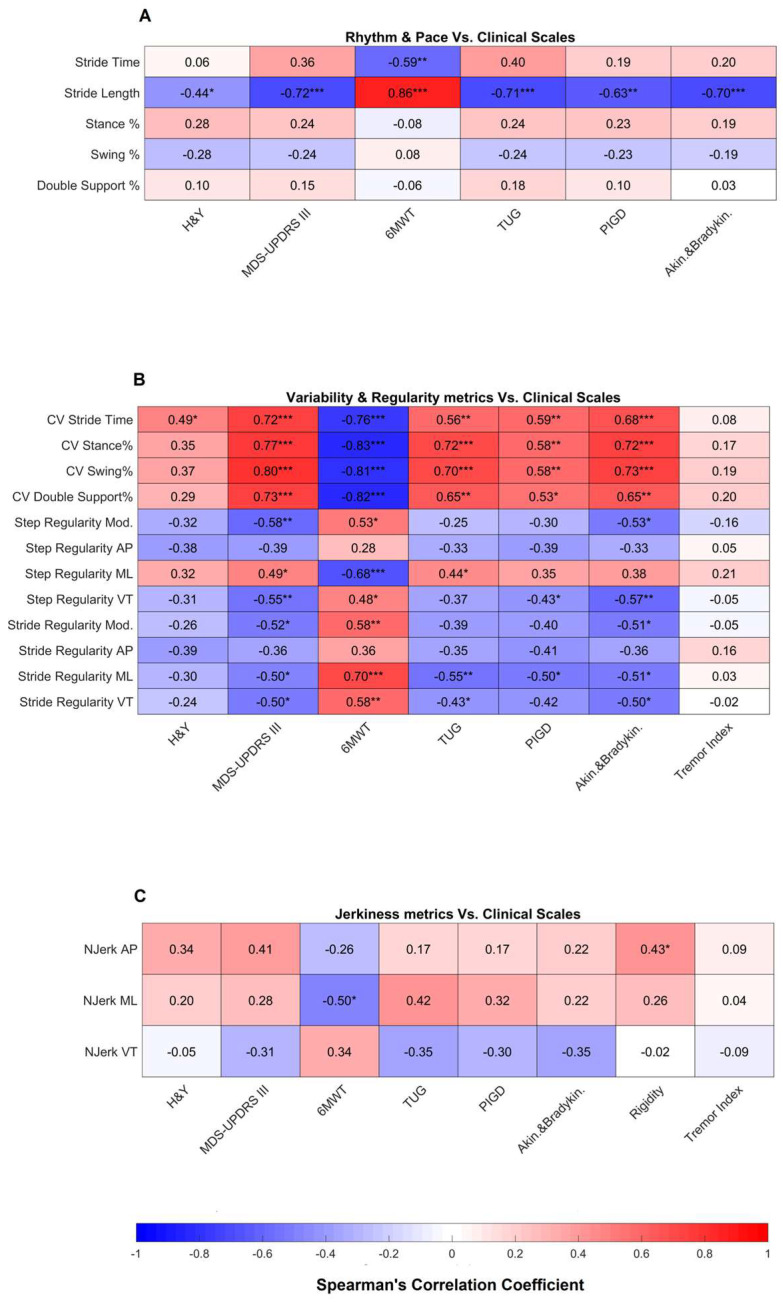
Correlation results of rhythm and pace (**A**), variability and regularity (**B**), and jerkiness (**C**) metrics vs. clinical scales. * indicates a *p*-value lower than 0.05, ** indicates a *p*-value lower than 0.01, *** indicates a *p*-value lower than 0.001. H&Y: Hoehn and Yahr; MDS-UPDRS: Movement Disorder Society—Unified Parkinson’s Disease Rating Scale; 6MWT: 6-Minute Walk Test; TUG: Timed Up and Go; PIGD: Postural Instability and Gait Difficulty; AP: Anteroposterior; ML: Mediolateral; VT: Vertical; CV: Coefficient of Variation; Njerk: Normalized Jerk.

**Figure 4:**
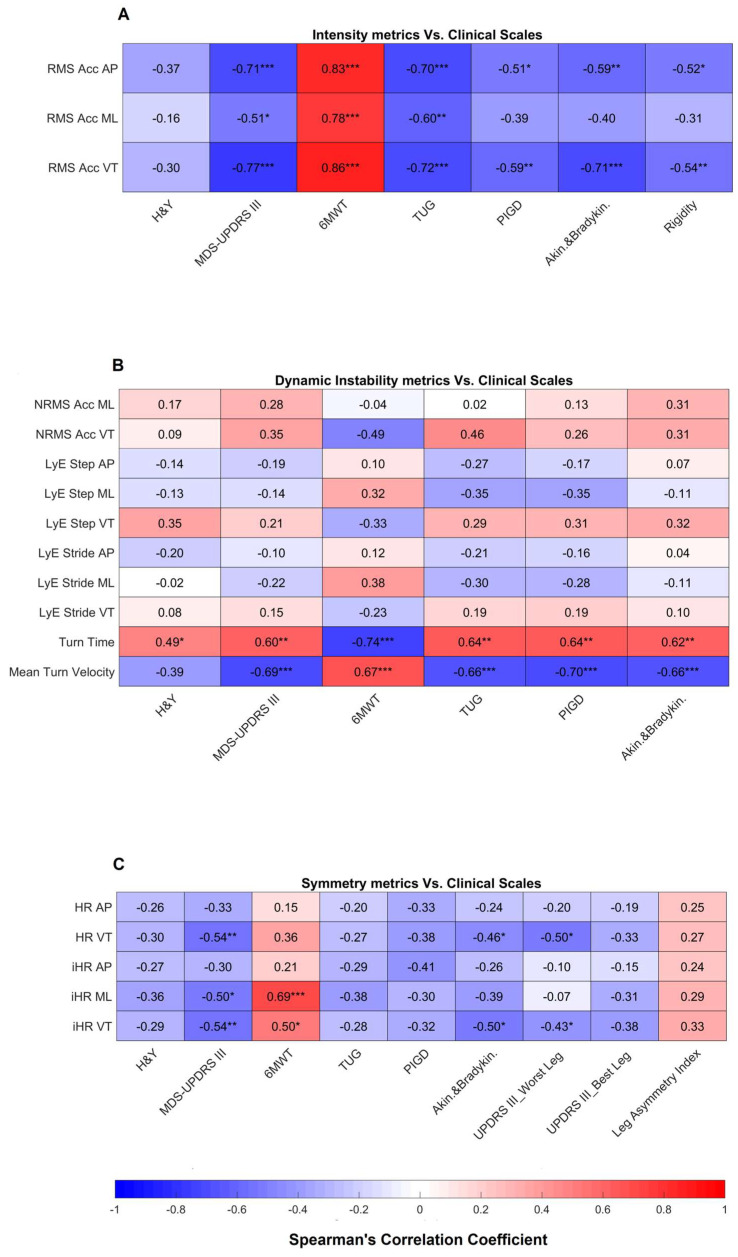
Correlations results of intensity (**A**), dynamic instability (**B**), and symmetry (**C**) metrics vs. clinical scales. * indicates a *p*-value lower than 0.05, ** indicates a *p*-value lower than 0.01, *** indicates a *p*-value lower than 0.001. H&Y: Hoehn and Yahr; MDS-UPDRS: Movement Disorder Society—Unified Parkinson’s Disease Rating Scale; 6MWT: 6-Minute Walk Test; TUG: Timed Up and Go; PIGD: Postural Instability and Gait Difficulty; RMS: Root Mean Square; AP: Anteroposterior; ML: Mediolateral; VT: Vertical; NRMS: Normalized Root Mean Square; LyE: Lyapunov Exponent; HR: Harmonic Ratio; iHR: improved Harmonic Ratio.

Results related to the correlation between highly reliable gait metrics and clinical scales are shown in Figure 3 and Figure 4. 

Regarding the rhythm and pace domain (Figure 3A), stride length showed a moderate to strong correlation with motor impairment (mH&Y and MDS-UPDRS III) and gait and balance measures (6MWT distance, TUG, PIGD and akinesia/bradykinesia). Similarly, variability and regularity metrics (Figure 3B) had an overall moderate correlation with MDS-UPDRS III and gait and balance scales. On the other hand, no correlation was found with the tremor index. In the jerkiness domain (Figure 3C), AP and ML normalized jerk correlated, respectively, with the rigidity index and the 6MWT distance. 

Moving to the intensity domain (Figure 4A), except for mH&Y all intensity metrics in AP and VT directions correlated with all outcome measures of gait and balance and of rigidity. In the ML direction, there was a correlation only with MDS-UPDRS III, 6MWT distance and TUG. Regarding the dynamic instability domain (Figure 4B), NRMS VT correlated with 6MWT and TUG, while turn time and mean turn velocity correlated with all motor impairment and gait and balance outcome measures. Conversely, no correlation was found between Lyapunov exponents and clinical scales. For symmetry metrics (Figure 4C), HR and iHR in the VT direction showed a correlation with MDS-UPDRS III, akinesia/bradykinesia and worst leg impairment, while no correlation was found with best leg impairment and leg asymmetry index. The iHR in the ML direction correlated only with MDS-UPDRS III and 6MWT. 

### 3.4. Discriminant Ability

Metrics that exhibited a statistically significant correlation with at least one clinical scale, hence showing concurrent validity, were further analyzed to determine their discriminant ability. The results are shown in Table 5.

After adjusting for multiple comparisons, eight metrics showed statistically significant differences between participants with mild PD and HS. In particular, compared to HS, people with PD walked with shorter stride length, reduced step regularity (as calculated from the module of the acceleration and the vertical component), lower vertical intensity and lower gait symmetry, as measured by HR and iHR in the vertical direction. Finally, turning was performed by people with PD with prolonged time and reduced angular velocity compared to HS.

Regarding AUC values, a good discriminant ability (AUC greater than 0.8) was identified for stride length, turn time, HR and iHR in vertical direction, while stance variability, step regularity calculated from module and vertical acceleration, vertical intensity and turning angular velocity showed a moderate discriminant ability (AUC between 0.7 and 0.8).

## 4. Discussion

In this work, forty-one metrics related to seven gait domains were extracted from lower trunk accelerations and angular velocities of twenty-two subjects with mild PD and ten healthy persons, while performing the 6MWT. The test–retest reliability assessed on participants with PD revealed good to excellent ICC values for 39/41 (95%) of the analyzed variables. The 59% (23/39) of reliable measures showed a moderate to strong correlation (concurrent validity) with at least one clinical scale. Finally, 8 out of 23 reliable and valid metrics (35%), were found statistically different between people with mild PD and HS, with a moderate to good discriminant ability. To the best of our knowledge, this is the first study analyzing these aspects in people with mild PD performing a 6MWT instrumented with a single IMU on the lower trunk.

### 4.1. Test–Retest Reliability

The results of the reliability assessment showed an overall high test–retest reliability for 95% of the investigated features, suggesting the possibility of using these metrics for the monitoring of disease progression or for assessing the effect of a pharmacological or rehabilitation treatment. In particular, all these metrics can be used for longitudinal group-level comparisons, since they showed ICC values greater or equal to the minimum acceptable threshold for this type of analysis (i.e., 0.75 [56]). In addition, 32 out of 39 reliable metrics (82%) showed ICC values ≥ 0.85, which is recommended for intra-individual comparisons [56]. These parameters included stride length and duration, all gait variability, regularity, jerkiness and intensity metrics, seven metrics descriptive of dynamic instability, e.g., turn time and angular velocity, and three metrics quantifying gait symmetry (i.e., HR VT, iHR AP, iHR VT). These parameters can therefore be used, not only for group-level analysis, but also for individual judgment and individual decision-making.

To the best of our knowledge, no published study exists assessing the test–retest reliability of instrumented metrics computed from a single inertial sensor during the 6MWT in people with PD. However, two studies [23,41] computed ICC to evaluate the agreement between a single IMU system and a reference system (i.e., electronic walkway) to measure spatiotemporal parameters of subjects with PD. Although these tests were carried out on shorter walks and with a different aim, the ICC values obtained by the authors are comparable to those here reported. Del Din et al. [41] extended their measurements by computing not only spatiotemporal parameters, but also variability and asymmetry metrics. Although our ICC results are similar to those obtained by del Din et al. for the spatiotemporal metrics, the variability and symmetry measures were different from those calculated in the present work, so a direct comparison is not possible. To our knowledge, there are no studies that determined the test–retest reliability of gait features belonging to other gait domains (i.e., jerkiness, intensity, dynamic stability) for people with PD. 

Considering different pathologies, such as multiple sclerosis and stroke, three works [19,20,21] focused on domains and metrics comparable to those here considered (e.g., variability, regularity, intensity and instability). Although data were acquired from a system of three IMUs (feet and lower trunk), our ICC values are comparable and slightly higher than those reported in these studies, suggesting that, from the reliability point of view, a single IMU can be used to further facilitate deployment in clinical practice. 

### 4.2. Concurrent Validity

All gait domains showed at least one statistically significant correlation between a reliable feature and a clinical scale, suggesting that each domain can be represented by at least one reliable and valid instrumented metric. 

Regarding pace and rhythm metrics, only stride length showed significant moderate to very strong correlations with all clinical scales assessing motor impairment and gait and balance. Coherently with other studies in the literature, stride length correlated with motor impairment measures (i.e., mH&Y and MDS-UPDRS III), as well as with PIGD, 6MWT, TUG and akinetic/bradykinetic signs [57,58,59,60], indicating that patients with more severe motor disorders, in particular in gait and balance, take shorter steps during walking. Regarding the other pace and rhythm metrics, stride time moderately correlated with the 6MWT only, while no correlation with clinical scales was found for stance, swing and double support duration. Taken together, these findings enforced previous studies that found a significant progressive decrease in stride length over the course of the disease [58,61], accompanied by minor changes in stride time and duration of stride sub-phases, which become evident only in more advanced stages of PD (i.e., mH&Y: 3–4) [58,61].

Regarding variability/regularity domain, our results are consistent with previous works showing significant positive correlations between gait variability and motor impairment as measured by H&Y and MDS-UPDRS III [62]. Variability measures correlated also with PIGD, TUG, 6MWT and akinetic/bradykinetic signs confirming previous results on persons with PD [57,63] and older adults [25], and highlighting the importance of gait variability to monitor the progression of gait and balance deficits [64]. Similarly, regularity variables showed relevant negative correlations with the MDS-UPDRS III scale, suggesting the progressive difficulty in regulating repeated strides during walking and controlling rhythmic movements, typical of PD. No correlation was found between the tremor index and the regularity and variability metrics, as found by Schaafsma et al. [65].

In the jerkiness domain, the normalized jerk in the ML direction showed a moderate negative correlation with walking endurance, as measured by the 6MWT. This is in line with the notion that lower jerk (higher smoothness) is associated with better motor control [66], and, consequently, higher walking endurance. Interestingly, normalized jerk in the AP direction was positively correlated to the rigidity index, indicating a progressive loss of gait smoothness in patients with increasing rigidity of body segments. Regarding this point, it can be speculated that, while healthy subjects walk with a “soft” heel strike and a smooth heel-to-toe pattern, people with Parkinson’s disease presenting rigidity of the trunk and lower limbs walk impacting the ground in a less dampened way and with a “flat-foot” gait characterized by reduced roll-off and, consequently, reduced smoothness [67,68]. 

As for the intensity domain, the RMS of the trunk acceleration components decreased with the worsening of motor symptoms. In particular, increasing rigidity and akinetic/bradykinetic signs are associated with smaller trunk sway, mainly in AP and VT directions [69]. Regarding gait and balance, lower amplitude of trunk accelerations was associated with lower walking endurance and poorer dynamic balance (i.e., higher PIGD and TUG [69]). This result is confirmed by a recent study, testing a cohort of persons with neurological diseases including PD, which found that, independently from gait speed, decreased trunk acceleration in the AP direction is strongly associated with decreased dynamic balance clinically measured by the modified Dynamic Gait Index (mDGI) [70]. Hence, as previously hypothesized [19], decreased trunk acceleration could be the result of minimizing upper body motion during walking trying to compensate for lower limb impairments and maintain balance.

Considering dynamic instability domain, turning duration and turning angular velocity confirmed to be good digital biomarkers of disease progression given their strong correlation with all selected clinical assessments, particularly with balance measures, as already found by previous works [33,69,71]. Unexpectedly, Lyapunov exponents (LyE) did not show significant correlations with any clinical evaluations, including balance measures (i.e., PIGD and TUG). This is in contrast with previous results on a group of people with PD, multiple sclerosis and stroke showing a significant association between increasing LyE Step AP and decreased balance as measured by the mDGI [70]. This can be due to the fact that our sample was composed of persons with PD who showed less severe walking impairment, as suggested by the 6MWT distance which was significantly higher (+156 m) compared to that characterizing the PD group tested by Liuzzi et al. [70]. Another possible explanation could be that, in the present study, LyE was computed over the duration of one step and stride. Based on the findings of Fino et al. [72], better results could have been obtained by calculating LyE over different stride periods, such as weight acceptance, early swing and mid swing phases.

Finally, regarding symmetry domain, HR and iHR (measuring gait symmetry) significantly correlated with motor impairment (MDS-UPDRS III). While no correlation was found with balance scales, iHR in the mediolateral and vertical directions was significantly associated with walking endurance (6MWT). This suggests that gait symmetry is primarily associated with energy efficiency of locomotion rather than balance [70,73]. In addition, no HRs showed significant correlations with Leg Asymmetry Index calculated from the specific MDS-UPDRS III items. This was partly expected since HRs and the clinical Leg Asymmetry Index measure different constructs: HRs quantified step-to-step symmetry during walking, which is a functional task, while the clinical assessment of asymmetry through MDS-UPDRS III implies the observation of rest positions, passive movements and non-functional active movements of feet and toes in sitting position. Despite this result, vertical HR and iHR showed a moderate and statistically significant negative correlation with the impairment of the most affected leg and a low non-significant correlation with the alterations of the less affected leg. This different behavior, in turn, suggests that gait symmetry is associated, at least partially, with the different levels of impairment between lower limbs.

### 4.3. Discriminant Ability

The statistical analysis via Mann–Whitney U test and AUC revealed significant differences between healthy subjects and people with mild PD in eight instrumented metrics showing a moderate–high discriminant ability.

In particular, the results confirmed the reduced stride length of subjects with PD when compared with healthy controls [74], while no significant alterations were found for cadence. The previous literature showed that PD causes higher difficulty in the regulation of stride length, while it maintains cadence control intact and adaptable [75,76]. Moreover, the difference in the RMS of VT acceleration between healthy and PD individuals was statistically significant. This result could be related to the found difference in stride length, since the RMS of acceleration has a close association with walking speed [77] and it is known that persons with PD walk slower through a reduced stride length [74].

A statistically significant lower step regularity (computed from the acceleration modulus and the VT component) was found in people with mild PD compared to HS, suggesting that this metric is an index of inconsistent and less rhythmic stepping patterns, which worsen with the progression of the disease [78].

In addition, turning time and turning velocity showed significant differences compared with healthy controls. Difficulty in turning is a common motor impairment that leads to longer turn time, more turn steps and lower turn velocities, increasing instability in people with PD [79,80,81].

Finally, we observed a less symmetric gait pattern through a significantly lower HR and iHR in the VT direction. Although previous works found significant alterations also in the AP and ML directions, our result confirms that subjects with mild PD are characterized by a higher gait asymmetry and less smooth movements compared to healthy people [45,82,83].

### 4.4. Study Limitations

Despite the found high reliability and validity of the gait features here analyzed, this study has some limitations. First, the PD group includes subjects with mild PD, thus the application of the present results to more severely affected patients must be carried out with caution. Secondly, the number of persons in the control group is too low to permit the determination of robust cutoffs that can help the clinician understand if the analyzed feature belongs to a healthy range. Finally, although this work provides MDCs that distinguish a real change from any casual variations, it is not possible to define if such real change is clinically significant. 

For the above reasons, further studies should include subjects with moderate to severe PD, increment the number of healthy individuals in the control group, and compute the minimally important clinical difference. 

## 5. Conclusions

This study examined the reliability, validity and discriminant ability of gait metrics obtained from a single IMU positioned on the lower back during the execution of the 6MWT by individuals with mild PD. Most of the instrumented features exhibited high reliability in the test–retest analysis, indicating the absence of large variations in the parameters between test and retest sessions. For each gait domain, several significant correlations with clinical scales were found, suggesting the validity of most instrumented metrics to assess walking in PD. Finally, eight reliable and valid metrics were characterized by a moderate/high ability to discriminate between healthy subjects and individuals with mild PD.

Based on the achieved results, examined metrics can be suitable for objectively and reliably assessing gait, and can be integrated into clinical practice to complement clinical evaluations [84] and to investigate longitudinal changes in subjects with mild PD to monitor the progression of the disease and/or the effects of a pharmacological/rehabilitation treatment.

## Figures and Tables

**Table 1:** Clinical scales and tests.

Outcome	Outcome Measure	Description
Motor Impairment	Modified Hoehn and Yahr (mH&Y)	mH&Y evaluates the staging of the functional disability due to PD, from 1 (unilateral involvement only) to 5 (wheelchair-bound or bedridden) [28,31].
	Movement Disorders Society—Unified Parkinson’s Disease Rating Scale Part 2 (MDS-UPDRS II)	Self-administered questionnaire focused on difficulties in 13 motor aspects of daily living. Each item is rated from 0 (normal) to 4 (severe) [32].
	Movement Disorders Society—Unified Parkinson’s Disease Rating Scale Part 3 (MDS-UPDRS III)	Assessment of motor signs of PD through the evaluation of 18 aspects rated by the examiner between 0 (normal) and 4 (severe) [32].
Gait and Balance	6-Minute Walk Test (6MWT)	Assessment of walking endurance by measuring the distance walked on a flat hard surface in 6 min [5].
	Timed Up and Go (TUG)	Assessment of mobility and dynamic balance [33]. It requires subjects to rise from a chair, walk 3 m, turn, walk back to the chair and sit down. The score is represented by the time taken to perform the test.
	Postural Instability and Gait Difficulty (PIGD)	Index defined by the mean scores of the MDS-UPDRS items 2.12, 2.13, 3.10, 3.11, 3.12 [34].
Asymmetry	Worst Leg and Best Leg Impairments	Indices derived by summing the MDS-UPDRS III scores related to right and left lower limb (items 3.3, 3.7, 3.8, 3.17). The highest (lowest) index quantifies the worst leg (best leg) impairment.
	Leg Asymmetry Index	Ratio between the difference of the worst and best leg, and the sum of the worst and best leg [35].
Akinesia/ Bradykinesia	Akinesia/Bradykinesia Index	Index evaluating the loss/slowness of spontaneous voluntary limb movement. It is derived by adding the MDS-UPDRS III scores of items from 3.4 to 3.11, 3.13 and 3.14 [36].
Rigidity	Rigidity index	Index defined by summing MDS-UPDRS III scores of item 3.3.
Tremor	Tremor index	Index defined by the mean scores of the MDS-UPDRS items 2.10, 3.15, 3.16, 3.17 and 3.18 [34].
Cognitive Impairment	Montreal Cognitive Assessment (MoCA)	MoCA consists of 16 items assessing different cognitive domains, including visuospatial and executive functions, memory, attention and orientation). The maximum score is 30 points indicating normal cognition. In the present paper, MoCA scores adjusted following Santangelo et al. [28] were also reported.

PD: Parkinson’s Disease.

**Table 2:** Summary of domains and instrumented metrics.

Domain	Metric	Description
Rhythm and Pace	Stride duration [s]	Time interval between two consecutive heel-strike of the same foot.
	Stance duration (%)	Time interval between the instants of heel-strike and toe-off of the same foot, expressed as a percentage of the stride time.
	Swing duration (%)	Time interval between the instants of toe-off of the current footfall and heel-strike of the next footfall of the same foot, expressed in percentage of the stride time.
	Double support duration (%)	Time interval between the instants of heel-strike of one foot and the toe-off of the contralateral foot, expressed in percentage of the stride time. For this paper, the sum of double supports of both legs was considered.
	Stride length [m]	Distance between two consecutive heel-strike of the same foot, calculated as the path length divided by the number of strides.
Variability	Coefficient of variation (CV) of stride duration, stance and swing duration, single and double support duration [-]	Ratio of the standard deviation of a parameter to the mean of the same parameter [45].
Regularity	Step and Stride regularity (Module, AP, ML, VT) [-]	Respectively, the first and the second peaks of the normalized autocorrelation function computed from each acceleration component and modulus. Increasing values, from 0 to 1, denote higher regularity between steps and strides [46].
Jerkiness	Normalized Jerk AP, ML, VT [-] (NJerk)	Jerk (first-time derivative of acceleration) normalized with respect to stride duration and mean acceleration. Higher values indicate less smooth movement [47].
Intensity	RMS Acc AP, ML, VT [m/s^2^]	RMS of each acceleration component. Decreasing values represent a higher capability for controlling postural control of the upper body [19].
Dynamic Instability	Normalized RMS Acc AP, ML, VT [-] (NRMS)	Ratio of the RMS of acceleration in a given direction to the RMS of acceleration magnitude [48].
	Lyapunov Exponent (LyE) for stride and step (AP, ML, VT) [-]	Exponent estimated from each acceleration following Rosenstein method [49]. A detailed description of LyE computation is reported elsewhere [50]. Increasing values indicate a higher sensitivity to small perturbations, reflecting lower dynamic stability.
	Turn time [s]	Time interval between the end of a straight path and the beginning of the next.
	Turning angular velocity [deg/s]	Mean angular velocity around the vertical axis during turns.
Symmetry	Harmonic Ratio—HR (AP, ML, VT) [-]	Ratio between the sum of the amplitudes of the in-phase harmonics and the sum of the amplitudes of the out-of-phase harmonics [45].
	Improved Harmonic Ratio—iHR (AP, ML, VT) [-]	Ratio between the power of the in-phase harmonics and the sum of in-phase and out-of-phase harmonics power [51].

RMS: Root Mean Square.

**Table 3:** Demographic and clinical characteristics of participants.

	PwPD (n = 22)	HS (n = 10)	*p*-Value
Age [years]	72.5 (63; 79.9)	70.5 (65.5; 76.1)	0.51
Sex [female/male]	8/14	6/4	0.21
Body Mass Index (BMI) [kg/m^2^]	24.7 (19.2; 31.6)	24.3 (20.9; 27.6)	0.73
mH&Y stage	2 (1.5; 2.5)	-	-
MDS-UPDRS II	6 (1.1; 19)	-	-
MDS-UPDRS III	31 (13.2; 42.9)	-	-
6MWT [m]	488 (262.2; 578.8)	565.8 (487.4; 616.7)	0.02 *
TUG [s]	9.5 (6.7; 16.8)	7.8 (6.7; 11.8)	0.16
PIGD	0.5 (0; 1)	-	-
Akinesia/Bradykinesia	19 (5.3; 29.9)	-	-
Most/least affected side (right/left)	10/12	-	-
Leg Asymmetry Index	0.3 (0.1; 0.9)	-	-
Rigidity index	3 (1; 7.9)	-	-
Tremor index	0.2 (0; 0.8)	-	-
MoCA	24 (20; 28)	-	-
Adjusted MoCA score	23.0 (19.6; 27.5)	-	-

Values are median (5th percentile; 95th percentile) or numbers. * indicates a *p*-value lower than 0.05. PwPD: People with Parkinson’s Disease; HS: Healthy Subjects; mH&Y: modified Hoehn and Yahr; MDS-UPDRS: Movement Disorder Society—Unified Parkinson’s Disease Rating Scale; 6MWT: 6-Minute Walk Test; TUG: Timed Up and Go; PIGD: Postural Instability and Gait Difficulty; MoCA: Montreal Cognitive Assessment.

**Table 4:** Test–retest reliability results.

Domains	Test Mean (SD)	Retest Mean (SD)	ICC	95% CI		SEM	MDC
				Lower Limit	Upper Limit		
**Rhythm and Pace**							
Stride duration [s]	0.95 (0.08)	0.95 (0.09)	0.98	0.94	0.99	1.28	3.54
Stance [%]	62.96 (1.06)	63.27 (1.41)	0.75	0.42	0.90	0.61	1.7
Swing [%]	37.04 (1.06)	36.73 (1.41)	0.75	0.42	0.90	0.61	1.7
Double support [%]	26.04 (1.94)	26.63 (2.57)	0.89	0.72	0.96	0.75	2.07
Stride length [m]	1.40 (0.24)	1.37 (0.23)	0.98	0.94	0.99	0.04	0.10
**Variability**							
Stride duration [-]	1.97 (0.82)	2.09 (0.90)	0.93	0.83	0.97	0.23	0.64
Stance [-]	1.87 (0.75)	1.90 (0.87)	0.95	0.89	0.98	0.17	0.48
Swing [-]	3.20 (1.30)	3.27 (1.48)	0.95	0.89	0.98	0.30	0.83
Single support [-]	1.65 (0.93)	1.80 (1.18)	0.95	0.87	0.98	0.24	0.67
Double support [-]	4.87 (2.67)	5.27 (3.09)	0.96	0.91	0.98	0.56	1.56
**Regularity**							
Step module [-]	0.80 (0.08)	0.80 (0.11)	0.90	0.76	0.96	0.03	0.08
Step AP [-]	0.55 (0.19)	0.54 (0.20)	0.89	0.74	0.96	0.06	0.18
Step ML [-]	−0.59 (0.15)	−0.59 (0.17)	0.90	0.76	0.96	0.05	0.14
Step VT [-]	0.79 (0.09)	0.77 (0.13)	0.90	0.76	0.96	0.03	0.10
Stride module [-]	0.86 (0.07)	0.84 (0.08)	0.92	0.79	0.97	0.02	0.06
Stride AP [-]	0.70 (0.13)	0.68 (0.12)	0.86	0.67	0.94	0.05	0.13
Stride ML [-]	0.71 (0.10)	0.69 (0.10)	0.91	0.77	0.96	0.03	0.09
Stride VT [-]	0.85 (0.08)	0.83 (0.09)	0.92	0.79	0.97	0.02	0.07
**Jerkiness**							
Njerk AP [-]	0.44 (0.11)	0.45 (0.11)	0.89	0.73	0.95	0.04	0.10
Njerk ML [-]	0.35 (0.06)	0.35 (0.05)	0.91	0.79	0.96	0.02	0.04
Njerk VT [-]	0.10 (0.03)	0.10 (0.02)	0.93	0.84	0.97	0.01	0.02
**Intensity**							
RMS Acc AP [m/s^2^]	2.26 (0.99)	2.36 (1.25)	0.93	0.84	0.97	0.29	0.80
RMS Acc ML [m/s^2^]	2.52 (1.29)	2.55 (1.46)	0.99	0.97	1.00	0.14	0.40
RMS Acc VT [m/s^2^]	3.12 (0.95)	3.09 (1.01)	0.96	0.90	0.98	0.20	0.54
**Dynamic Instability**							
NRMS Acc AP [-]	0.65 (0.10)	0.68 (0.17)	0.73	0.35	0.89	0.07	0.19
NRMS Acc ML [-]	0.72 (0.16)	0.72 (0.17)	0.96	0.90	0.98	0.03	0.09
NRMS Acc VT [-]	0.92 (0.08)	0.92 (0.08)	0.95	0.88	0.98	0.02	0.05
LyE Step AP [-]	0.64 (0.14)	0.69 (0.17)	0.84	0.61	0.93	0.06	0.17
LyE Step ML [-]	0.71 (0.19)	0.77 (0.23)	0.93	0.72	0.97	0.06	0.16
LyE Step VT [-]	0.80 (0.15)	0.83 (0.15)	0.87	0.68	0.95	0.05	0.15
LyE Stride AP [-]	0.44 (0.09)	0.47 (0.12)	0.80	0.54	0.92	0.05	0.13
LyE Stride ML [-]	0.47 (0.14)	0.52 (0.17)	0.92	0.76	0.97	0.04	0.12
LyE Stride VT [-]	0.56 (0.10)	0.59 (0.11)	0.82	0.58	0.93	0.04	0.12
Turn time [s]	2.71 (1.89)	3.02 (2.79)	0.96	0.90	0.98	0.49	1.36
Turning velocity [deg/s]	13.67 (3.19)	13.96 (3.01)	0.94	0.85	0.97	0.78	2.16
**Symmetry**							
HR AP [-]	1.97 (0.46)	1.94 (0.45)	0.77	0.43	0.90	0.22	0.60
HR ML [-]	2.29 (0.28)	2.36 (0.46)	0.64	0.15	0.85	0.22	0.62
HR VT [-]	2.74 (0.52)	2.76 (0.59)	0.91	0.78	0.96	0.17	0.46
Improved HR AP [-]	76.50 (8.09)	75.59 (8.99)	0.89	0.73	0.95	2.89	8.02
Improved HR ML [-]	80.98 (4.49)	81.16 (6.05)	0.84	0.61	0.93	2.11	5.84
Improved HR VT [-]	88.89 (5.12)	88.39 (6.16)	0.93	0.84	0.97	1.47	4.07

AP: Anteroposterior; ML: Mediolateral; VT: Vertical; Njerk: Normalized Jerk; RMS: Root Mean Square; NRMS: Normalized Root Mean Square; LyE: Lyapunov Exponent; HR: Harmonic Ratio.

**Table 5:** Comparisons between people with mild PD (PwPD) and healthy subjects (HS).

Domains	Median (5th; 95th Percentile)		*p*-Value	Mean (95% CI)	
	PwPD	HS		AUC	
**Rhythm and Pace**					
Stride duration [s]	0.93 (0.86; 1.10)	0.93 (0.85; 1.00)	0.411	0.55 (0.34; 0.77)	
Stride length [m]	1.47 (0.94;1.69)	1.62 (1.48; 1.84)	0.002 *	0.83 (0.69; 0.97)	
**Variability**					
CV Stride duration [-]	1.81 (1.12; 3.62)	1.49 (1.65; 1.91)	0.119	0.67 (0.47; 0.86)	
CV Stance [-]	1.65 (1.06; 3.42)	1.29 (1.01; 1.70)	0.149	0.72 (0.54; 0.90)	
CV Swing [-]	2.83 (1.76; 5.67)	2.27 (1.83; 2.93)	0.168	0.67 (0.47; 0.86)	
CV Double support [-]	3.89 (2.55; 11.10)	3.37 (2.66; 4.42)	0.190	0.64 (0.44; 0.84)	
**Regularity**					
Step Reg. MOD [-]	0.83 (0.68; 0.90)	0.89 (0.82; 0.92)	0.024 *	0.76 (0.59; 0.93)	
Step Reg. ML [-]	−0.64 (−0.77; −0.29)	−0.73 (−0.81; −0.36)	0.279	0.65 (0.45; 0.85)	
Step Reg. VT [-]	0.81 (0.67; 0.90)	0.89 (0.80; 0.93)	0.037 *	0.78 (0.61; 0.94)	
Stride Reg. MOD [-]	0.88 (0.74; 0.94)	0.89 (0.82; 0.94)	0.366	0.61 (0.40; 0.82)	
Stride Reg. ML [-]	0.71 (0.56; 0.84)	0.80 (0.55; 0.85)	0.210	0.66 (0.46; 0.86)	
Stride Reg. VT [-]	0.87 (0.71; 0.94)	0.80 (0.55; 0.85)	0.411	0.59 (0.38; 0.80)	
**Jerkiness**					
Njerk AP [-]	0.41 (0.29; 0.58)	0.40 (0.30; 0.69)	0.562	0.57 (0.36; 0.78)	
Njerk ML [-]	0.35 (0.26; 0.43)	0.30 (0.25; 0.48)	0.704	0.64 (0.44; 0.84)	
**Intensity**					
RMS Acc AP [m/s^2^]	2.01 (1.22; 3.53)	2.96 (1.88; 2.96)	0.105	0.69 (0.51; 0.88)	
RMS Acc ML [m/s^2^]	2.30 (1.27; 3.86)	2.90 (1.82; 4.30)	0.140	0.64 (0.44; 0.84)	
RMS Acc VT [m/s^2^]	3.20 (1.86; 4.34)	4.05 (3.13; 4.71)	0.041 *	0.75 (0.58; 0.92)	
**Dynamic Instability**					
NRMS Acc VT [-]	0.95 (0.80; 0.98)	0.92 (0.87; 0.98)	0.345	0.61 (0.40; 0.81)	
Turn time [s]	2.18 (1.82; 3.96)	1.83 (1.69; 2.03)	0.002 *	0.82 (0.67; 0.97)	
Turning velocity [deg/s]	13.94 (8.78; 17.48)	15.42 (13.01; 18.01)	0.028 *	0.71 (0.52; 0.59)	
**Asymmetry**					
HR VT [-]	2.83 (1.92; 3.45)	3.77 (2.69; 4.23)	0.002 *	0.85 (0.72; 0.98)	
Improved HR ML [-]	82.53 (74.52; 87.36)	85.23 (69.66; 89.99)	0.305	0.62 (0.41; 0.82)	
Improved HR VT [-]	90.57 (80.25; 94.73)	94.43 (90.38; 95.77)	0.003 *	0.82 (0.67; 0.96)	

* indicates a *p*-value lower than 0.05. AP: Anteroposterior; ML: Mediolateral; VT: Vertical; CV: Coefficient of Variation; Njerk: Normalized jerk; RMS: Root Mean Square; NRMS: Normalized Root Mean Square; HR: Harmonic Ratio.

## Data Availability

The dataset used and/or analyzed during the current study is available from the corresponding author under reasonable request.

## References

[1] Balestrino R., Schapira A.H.V. (2020). Parkinson Disease. Eur. J. Neurol..

[2] García D.S., Fonticoba T.D.D., Bartolomé C.C., Ríos L.N., Roca L.G., Miró C.M., Canfield H., Jesús S., Aguilar M., Pastor P. (2021). Predictors of Loss of Functional Independence in Parkinson’s Disease: Results from the Coppadis Cohort at 2-Year Follow-up and Comparison with a Control Group. Diagnostics.

[3] Shulman L.M., Armstrong M., Ellis T., Gruber-Baldini A., Horak F., Nieuwboer A., Parashos S., Post B., Rogers M., Siderowf A. (2016). Disability Rating Scales in Parkinson’s Disease: Critique and Recommendations. Mov. Disord..

[4] Bloem B.R., Marinus J., Almeida Q., Dibble L., Nieuwboer A., Post B., Ruzicka E., Goetz C., Stebbins G., Martinez-Martin P. (2016). Measurement Instruments to Assess Posture, Gait, and Balance in Parkinson’s Disease: Critique and Recommendations. Mov. Disord..

[5] Crapo R.O., Casaburi R., Coates A.L., Enright P.L., MacIntyre N.R., McKay R.T., Johnson D., Wanger J.S., Zeballos R.J., Bittner V. (2002). ATS Statement: Guidelines for the Six-Minute Walk Test. Am. J. Respir. Crit. Care Med..

[6] Macchiavelli A., Giffone A., Ferrarello F., Paci M. (2021). Reliability of the Six-Minute Walk Test in Individuals with Stroke: Systematic Review and Meta-Analysis. Neurol. Sci..

[7] Ries J.D., Echternach J.L., Nof L., Blodgett M.G. (2009). Test-Retest Reliability and Minimal Detectable Change Scores for the Timed “up & Go” Test, the Six-Minute Walk Test, and Gait Speed in People with Alzheimer Disease. Phys. Ther..

[8] Goldman M.D., Marrie R.A., Cohen J.A. (2008). Evaluation of the Six-Minute Walk in Multiple Sclerosis Subjects and Healthy Controls. Mult. Scler. J..

[9] Steffen T., Seney M. (2008). Test-Retest Reliability and Minimal Detectable Change on Balance and Ambulation Tests, the 36-Item Short-Form Health Survey, and the Unified Parkinson Disease Rating Scale in People with Parkinsonism. Phys. Ther..

[10] Kobayashi E., Himuro N., Takahashi M. (2017). Clinical Utility of the 6-Min Walk Test for Patients with Moderate Parkinson’s Disease. Int. J. Rehabil. Res..

[11] Washabaugh E.P., Kalyanaraman T., Adamczyk P.G., Claflin E.S., Krishnan C. (2017). Validity and Repeatability of Inertial Measurement Units for Measuring Gait Parameters. Gait Posture.

[12] Cho Y.S., Jang S.H., Cho J.S., Kim M.J., Lee H.D., Lee S.Y., Moon S.B. (2018). Evaluation of Validity and Reliability of Inertial Measurement Unit-Based Gait Analysis Systems. Ann. Rehabil. Med..

[13] Flachenecker F., Gaßner H., Hannik J., Lee D.H., Flachenecker P., Winkler J., Eskofier B., Linker R.A., Klucken J. (2020). Objective Sensor-Based Gait Measures Reflect Motor Impairment in Multiple Sclerosis Patients: Reliability and Clinical Validation of a Wearable Sensor Device. Mult. Scler. Relat. Disord..

[14] Esser P., Dawes H., Collett J., Feltham M.G., Howells K. (2012). Validity and Inter-Rater Reliability of Inertial Gait Measurements in Parkinson’s Disease: A Pilot Study. J. Neurosci. Methods.

[15] Lencioni T., Meloni M., Bowman T., Marzegan A., Caronni A., Carpinella I., Castagna A., Gower V., Ferrarin M., Pelosin E. (2022). Events Detection of Anticipatory Postural Adjustments through a Wearable Accelerometer Sensor Is Comparable to That Measured by the Force Platform in Subjects with Parkinson’s Disease. Sensors.

[16] Riva F., Bisi M.C., Stagni R. (2014). Gait Variability and Stability Measures: Minimum Number of Strides and within-Session Reliability. Comput. Biol. Med..

[17] Storm F.A., Cesareo A., Reni G., Biffi E. (2020). Wearable Inertial Sensors to Assess Gait during the 6-Minute Walk Test: A Systematic Review. Sensors.

[18] Pires I.M., Villasana M.V., Sá J., Denysyuk H.V., Marques D.L., Morgado J.F., Albuquerque C., Zdravevski E. (2022). Development Technologies for the Monitoring of Six-Minute Walk Test: A Systematic Review. Sensors.

[19] Angelini L., Hodgkinson W., Smith C., Dodd J.M., Sharrack B., Mazzà C., Paling D. (2020). Wearable Sensors Can Reliably Quantify Gait Alterations Associated with Disability in People with Progressive Multiple Sclerosis in a Clinical Setting. J. Neurol..

[20] Angelini L., Carpinella I., Cattaneo D., Ferrarin M., Gervasoni E., Sharrack B., Paling D., Nair K.P.S., Mazzà C. (2020). Is a Wearable Sensor-Based Characterisation of Gait Robust Enough to Overcome Differences between Measurement Protocols? A Multi-Centric Pragmatic Study in Patients with Multiple Sclerosis. Sensors.

[21] Felius R.A.W., Geerars M., Bruijn S.M., van Dieën J.H., Wouda N.C., Punt M. (2022). Reliability of IMU-Based Gait Assessment in Clinical Stroke Rehabilitation. Sensors.

[22] De Ridder R., Lebleu J., Willems T., De Blaiser C., Detrembleur C., Roosen P. (2019). Concurrent Validity of a Commercial Wireless Trunk Triaxial Accelerometer System for Gait Analysis. J. Sport. Rehabil..

[23] Vítečková S., Horáková H., Poláková K., Krupička R., Růžička E., Brožová H. (2020). Agreement between the GAITRite R System and the Wearable Sensor BTS G-Walk R for Measurement of Gait Parameters in Healthy Adults and Parkinson’s Disease Patients. PeerJ.

[24] Zago M., Sforza C., Pacifici I., Cimolin V., Camerota F., Celletti C., Condoluci C., De Pandis M.F., Galli M. (2018). Gait Evaluation Using Inertial Measurement Units in Subjects with Parkinson’s Disease. J. Electromyogr. Kinesiol..

[25] Grimpampi E., Oesen S., Halper B., Hofmann M., Wessner B., Mazzà C. (2015). Reliability of Gait Variability Assessment in Older Individuals during a Six-Minute Walk Test. J. Biomech..

[26] Pollet J., Buraschi R., Villafañe J.H., Piovanelli B., Negrini S. (2021). Gait Parameters Assessed with Inertial Measurement Unit during 6-Minute Walk Test in People after Stroke. Int. J. Rehabil. Res..

[27] Postuma R.B., Berg D., Stern M., Poewe W., Olanow C.W., Oertel W., Obeso J., Marek K., Litvan I., Lang A.E. (2015). MDS Clinical Diagnostic Criteria for Parkinson’s Disease. Mov. Disord..

[28] Santangelo G., Siciliano M., Pedone R., Vitale C., Falco F., Bisogno R., Siano P., Barone P., Grossi D., Santangelo F. (2015). Normative Data for the Montreal Cognitive Assessment in an Italian Population Sample. Neurol. Sci..

[29] Steffen T.M., Hacker T.A., Mollinger L. (2002). Age- and Gender-Related Test Performance in Community-Dwelling Elderly People: Six-Minute Walk Test, Berg Balance Scale, Timed Up & Go Test, and Gait Speeds. Phys. Ther..

[30] Li L., Zeng L., Lin Z.-J., Cazzell M., Liu H. (2015). Tutorial on Use of Intraclass Correlation Coefficients for Assessing Intertest Reliability and Its Application in Functional Near-Infrared Spectroscopy-Based Brain Imaging. J. Biomed. Opt..

[31] Hoehn M.M., Yahr M.D. (1967). Parkinsonism: Onset, Progression, and Mortality. Neurology.

[32] Goetz C.G., Tilley B.C., Shaftman S.R., Stebbins G.T., Fahn S., Martinez-Martin P., Poewe W., Sampaio C., Stern M.B., Dodel R. (2008). Movement Disorder Society-Sponsored Revision of the Unified Parkinson’s Disease Rating Scale (MDS-UPDRS): Scale Presentation and Clinimetric Testing Results. Mov. Disord..

[33] Picardi M., Redaelli V., Antoniotti P., Pintavalle G., Aristidou E., Sterpi I., Meloni M., Corbo M., Caronni A. (2020). Turning and Sit-to-Walk Measures from the Instrumented Timed Up and Go Test Return Valid and Responsive Measures of Dynamic Balance in Parkinson’s Disease. Clin. Biomech..

[34] Stebbins G.T., Goetz C.G., Burn D.J., Jankovic J., Khoo T.K., Tilley B.C. (2013). How to Identify Tremor Dominant and Postural Instability/Gait Difficulty Groups with the Movement Disorder Society Unified Parkinson’s Disease Rating Scale: Comparison with the Unified Parkinson’s Disease Rating Scale. Mov. Disord..

[35] Plotnik M., Giladi N., Balash Y., Peretz C., Hausdorff J.M. (2005). Is Freezing of Gait in Parkinson’s Disease Related to Asymmetric Motor Function?. Ann. Neurol..

[36] Kann S.J., Chang C., Manza P., Leung H.C. (2020). Akinetic Rigid Symptoms Are Associated with Decline in a Cortical Motor Network in Parkinson’s Disease. NPJ Park. Dis..

[37] Pacini Panebianco G., Bisi M.C., Stagni R., Fantozzi S. (2018). Analysis of the Performance of 17 Algorithms from a Systematic Review: Influence of Sensor Position, Analysed Variable and Computational Approach in Gait Timing Estimation from IMU Measurements. Gait Posture.

[38] Nguyen H.P., Ayachi F., Lavigne-Pelletier C., Blamoutier M., Rahimi F., Boissy P., Jog M., Duval C. (2015). Auto Detection and Segmentation of Physical Activities during a Timed-Up-and-Go (TUG) Task in Healthy Older Adults Using Multiple Inertial Sensors. J. Neuroeng. Rehabil..

[39] Moe-Nilssen R. (1998). A New Method for Evaluating Motor Control in Gait under Real-Life Environmental Conditions. Part 1: The Instrument. Clin. Biomech..

[40] McCamley J., Donati M., Grimpampi E., Mazzà C. (2012). An Enhanced Estimate of Initial Contact and Final Contact Instants of Time Using Lower Trunk Inertial Sensor Data. Gait Posture.

[41] Del Din S., Godfrey A., Rochester L. (2016). Validation of an Accelerometer to Quantify a Comprehensive Battery of Gait Characteristics in Healthy Older Adults and Parkinson’s Disease: Toward Clinical and at Home Use. IEEE J. Biomed. Health Inform..

[42] Lord S., Galna B., Verghese J., Coleman S., Burn D., Rochester L. (2013). Independent Domains of Gait in Older Adults and Associated Motor and Nonmotor Attributes: Validation of a Factor Analysis Approach. J. Gerontol. Ser. A.

[43] Lord S., Galna B., Rochester L. (2013). Moving Forward on Gait Measurement: Toward a More Refined Approach. Mov. Disord..

[44] Buckley C., Galna B., Rochester L., Mazzà C. (2019). Upper Body Accelerations as a Biomarker of Gait Impairment in the Early Stages of Parkinson’s Disease. Gait Posture.

[45] Lowry K.A., Smiley-Oyen A.L., Carrel A.J., Kerr J.P. (2009). Walking Stability Using Harmonic Ratios in Parkinson’s Disease. Mov. Disord..

[46] Moe-Nilssen R., Helbostad J.L. (2004). Estimation of Gait Cycle Characteristics by Trunk Accelerometry. J. Biomech..

[47] Contreras-Vidal J.L., Buch E.R. (2003). Effects of Parkinson’s Disease on Visuomotor Adaptation. Exp. Brain Res..

[48] Sekine M., Tamura T., Yoshida M., Suda Y., Kimura Y., Miyoshi H., Kijima Y., Higashi Y., Fujimoto T. (2013). A Gait Abnormality Measure Based on Root Mean Square of Trunk Acceleration. J. Neuroeng. Rehabil..

[49] Rosenstein M.T., Collins J.J., De Luca C.J. (1992). A Practical Method. for Calculating Largest Lyapunov Exponents from Small Data Sets. Phys. D Nonlinear Phenom..

[50] Caronni A., Gervasoni E., Ferrarin M., Anastasi D., Brichetto G., Confalonieri P., Giovanni R.D.I., Prosperini L., Tacchino A., Solaro C. (2020). Local Dynamic Stability of Gait in People with Early Multiple Sclerosis and No-to-Mild Neurological Impairment. IEEE Trans. Neural Syst. Rehabil. Eng..

[51] Pasciuto I., Bergamini E., Iosa M., Vannozzi G., Cappozzo A. (2017). Overcoming the Limitations of the Harmonic Ratio for the Reliable Assessment of Gait Symmetry. J. Biomech..

[52] Almarwani M., Perera S., VanSwearingen J.M., Sparto P.J., Brach J.S. (2016). The Test-Retest Reliability and Minimal Detectable Change of Spatial and Temporal Gait Variability during Usual over-Ground Walking for Younger and Older Adults. Gait Posture.

[53] Campbell M.J., Swinscow T.D.V., Thomas D.V. (2009). Statistics at Square One.

[54] Mcdonald J.H. (2009). Handbook of Biological Statistics.

[55] de Hond A.A.H., Steyerberg E.W., van Calster B. (2022). Interpreting Area under the Receiver Operating Characteristic Curve. Lancet Digit. Health.

[56] Streiner D.L., Norman G.R., Cairney J. (2014). Health Measurement Scales: A Practical Guide to Their Development and Use. Case Stud. Clin. Psychol. Sci. Bridg. Gap Sci. Pract..

[57] Sidoroff V., Raccagni C., Kaindlstorfer C., Eschlboeck S., Fanciulli A., Granata R., Eskofier B., Seppi K., Poewe W., Willeit J. (2021). Characterization of Gait Variability in Multiple System Atrophy and Parkinson’s Disease. J. Neurol..

[58] Schlachetzki J.C.M., Barth J., Marxreiter F., Gossler J., Kohl Z., Reinfelder S., Gassner H., Aminian K., Eskofier B.M., Winkler J. (2017). Wearable Sensors Objectively Measure Gait Parameters in Parkinson’s Disease. PLoS ONE.

[59] Clavijo-Buendía S., Molina-Rueda F., Martín-Casas P., Ortega-Bastidas P., Monge-Pereira E., Laguarta-Val S., Morales-Cabezas M., Cano-de-la-Cuerda R. (2020). Construct Validity and Test-Retest Reliability of a Free Mobile Application for Spatio-Temporal Gait Analysis in Parkinson’s Disease Patients. Gait Posture.

[60] Nieuwboer A., De Weerdt W., Dom R., Lesaffre E. (1998). A Frequency and Correlation Analysis of Motor Deficits in Parkinson Patients. Disabil. Rehabil..

[61] Welzel J., Wendtland D., Warmerdam E., Romijnders R., Elshehabi M., Geritz J., Berg D., Hansen C., Maetzler W. (2021). Step Length Is a Promising Progression Marker in Parkinson’s Disease. Sensors.

[62] Hausdorff J.M., Cudkowicz M.E., Firtion R., Wei J.Y., Goldberger A.L. (1998). Gait Variability and Basal Ganglia Disorders: Stride-to-Stride Variations of Gait Cycle Timing in Parkinson’s Disease and Huntington’s Disease. Mov. Disord..

[63] Rennie L., Dietrichs E., Moe-Nilssen R., Opheim A., Franzén E. (2017). The Validity of the Gait Variability Index for Individuals with Mild to Moderate Parkinson’s Disease. Gait Posture.

[64] Hobert M.A., Nussbaum S., Heger T., Berg D., Maetzler W., Heinzel S. (2019). Progressive Gait Deficits in Parkinson’s Disease: A Wearable-Based Biannual 5-Year Prospective Study. Front. Aging Neurosci..

[65] Schaafsma J.D., Giladi N., Balash Y., Bartels A.L., Gurevich T., Hausdorff J.M. (2003). Gait Dynamics in Parkinson’s Disease: Relationship to Parkinsonian Features, Falls and Response to Levodopa. J. Neurol. Sci..

[66] Dasgupta P. (2021). Acceleration Signals in Determining Gait-Related Difficulties and the Motor Skill of Walking in Older Adults. Ph.D. Thesis.

[67] Baltadjieva R., Giladi N., Gruendlinger L., Peretz C., Hausdorff J.M. (2006). Marked Alterations in the Gait Timing and Rhythmicity of Patients with de Novo Parkinson’s Disease. Eur. J. Neurosci..

[68] Hughes J., Bowes S., Leeman A., O’Neill C., Deshmukh A., Nicholson P., Dobbs S., Dobbs R. (1990). Parkinsonian Abnormality of Foot Strike: A Phenomenon of Ageing and/or One Responsive to Levodopa Therapy?. Br. J. Clin. Pharmacol..

[69] Palmerini L., Mellone S., Avanzolini G., Valzania F., Chiari L. (2013). Quantification of Motor Impairment in Parkinson’s Disease Using an Instrumented Timed up and Go Test. IEEE Trans. Neural Syst. Rehabil. Eng..

[70] Liuzzi P., Carpinella I., Anastasi D., Gervasoni E., Lencioni T., Bertoni R., Carrozza M.C., Cattaneo D., Ferrarin M., Mannini A. (2023). Machine Learning Based Estimation of Dynamic Balance and Gait Adaptability in Persons with Neurological Diseases Using Inertial Sensors. Sci. Rep..

[71] Zampieri C., Salarian A., Carlson-Kuhta P., Aminian K., Nutt J.G., Horak F.B. (2010). The Instrumented Timed Up and Go Test: Potential Outcome Measure for Disease Modifying Therapies in Parkinson’s Disease. J. Neurol. Neurosurg. Psychiatry.

[72] Fino P.C., Mancini M., Curtze C., Nutt J.G., Horak F.B. (2018). Gait Stability Has Phase-Dependent Dual-Task Costs in Parkinson’s Disease. Front. Neurol..

[73] Ellis R.G., Howard K.C., Kram R. (2013). The Metabolic and Mechanical Costs of Step Time Asymmetry in Walking. Proc. R. Soc. B Biol. Sci..

[74] Morris M.E., Iansek R., Matyas T.A., Summers J.J. (1996). Stride Length Regulation in Parkinson’s Disease. Normalization Strategies and Underlying Mechanisms. Brain.

[75] Morris M.E., Iansek R., Matyas T.A., Summers J.J. (1994). Ability to Modulate Walking Cadence Remains Intact in Parkinson’s Disease. J. Neurol. Neurosurg. Psychiatry.

[76] Bayle N., Patel A.S., Crisan D., Guo L.J., Hutin E., Weisz D.J., Moore S.T., Gracies J.M. (2016). Contribution of Step Length to Increase Walking and Turning Speed as a Marker of Parkinson’s Disease Progression. PLoS ONE.

[77] Menz H.B., Lord S.R., Fitzpatrick R.C. (2003). Acceleration Patterns of the Head and Pelvis When Walking on Level and Irregular Surfaces. Gait Posture.

[78] Herman T., Weiss A., Brozgol M., Giladi N., Hausdorff J.M. (2014). Gait and Balance in Parkinson’s Disease Subtypes: Objective Measures and Classification Considerations. J. Neurol..

[79] Stack E., Ashburn A. (2008). Dysfunctional Turning in Parkinson’s Disease. Disabil. Rehabil..

[80] Salarian A., Zampieri C., Horak F.B., Carlson-Kuhta P., Nutt J.G., Aminian K. Analyzing 180° Turns Using an Inertial System Reveals Early Signs of Progress in Parkinson’s Disease. Proceedings of the Annual International Conference of the IEEE Engineering in Medicine and Biology Society.

[81] Visser J.E., Voermans N.C., Nijhuis L.B.O., van der Eijk M., Nijk R., Munneke M., Bloem B.R. (2007). Quantification of Trunk Rotations during Turning and Walking in Parkinson’s Disease. Clin. Neurophysiol..

[82] Latt M.D., Menz H.B., Fung V.S., Lord S.R. (2009). Acceleration Patterns of the Head and Pelvis During Gait in Older People with Parkinson’s Disease: A Comparison of Fallers and Nonfallers. J. Gerontol. Ser. A.

[83] Sejdic E., Lowry K.A., Bellanca J., Redfern M.S., Brach J.S. (2014). A Comprehensive Assessment of Gait Accelerometry Signals in Time, Frequency and Time-Frequency Domains. IEEE Trans. Neural Syst. Rehabil. Eng..

[84] Hill E.J., Mangleburg C.G., Alfradique-Dunham I., Ripperger B., Stillwell A., Saade H., Rao S., Fagbongbe O., von Coelln R., Tarakad A. (2021). Quantitative Mobility Measures Complement the MDS-UPDRS for Characterization of Parkinson’s Disease Heterogeneity. Park. Relat. Disord..

